# On Carleson’s inequality

**DOI:** 10.1186/s13660-018-1682-2

**Published:** 2018-04-19

**Authors:** Ern Gun Kwon

**Affiliations:** Department of Mathematics-Education, Andong National University, Andong, Korea

**Keywords:** 26D10, 26A15, 26A51, Hardy inequality, Carleson’s inequality, Polya–Knopp inequality

## Abstract

We present a new proof of Hardy’s inequality by giving an $L^{p}$ version of Carleson’s inequality.

## Introduction

The classical Hardy inequality reads

1.1 $$ \int_{0}^{\infty}\biggl( \frac{1}{x} \int_{0}^{x} f(y) \,dy \biggr)^{p} \,dx \leq \biggl( \frac{p}{p-1} \biggr)^{p} \int_{0}^{\infty}f^{p}(x) \,dx, $$

where *f* is a nonnegative measurable function on $(0, \infty)$ and $p>1$. A weighted modification of (1.1) was proved also by Hardy [1]:

1.2 $$ \int_{0}^{\infty}\biggl( \frac{1}{x} \int_{0}^{x} f(y) \,dy \biggr)^{p} x^{\alpha}{\,dx} \leq \biggl( \frac{p}{p-1-\alpha} \biggr)^{p} \int_{0}^{\infty}f^{p}(x) x^{\alpha}\,dx, $$

where *f* is a nonnegative measurable function on $(0, \infty)$ and $p>1$, $\alpha< p-1$. The constant

$$\biggl( \frac{p}{p-1-\alpha} \biggr)^{p} $$

is the best possible.

The importance and the usefulness of these inequalities could never have been overestimated. Connected with Hardy’s inequality, the following two inequalities frequently appeared in the literature. See [2, 3], and [4].

### Theorem 1.1

(Polya–Knopp inequality)

1.3 $$ \int_{0}^{\infty}\exp \biggl\{ \frac{1}{x} \int_{0}^{x} \ln f(t) \,dt \biggr\} \,dx \leq e \int_{0}^{\infty}f(x) \,dx $$

*for any measurable* $f \ge0$.

### Theorem 1.2

(Carleson’s inequality)

*Let* $F(x)$ *be a convex function for* $x \geq0$, *satisfying* $F(0)=0$. *If* $-1<\alpha<\infty$, *the following inequality holds true*:

1.4 $$ \int_{0}^{\infty}x^{\alpha}e^{-F(x)/x} \,dx \leq e^{\alpha +1} \int_{0}^{\infty}x^{\alpha}e^{-F'(x)} \,dx. $$

As we shall see in Sect. 3.2, the following can be derived from (1.4) for any measurable $f\ge0$:

1.5 $$ \int_{0}^{\infty}x^{\alpha}\exp \biggl\{ \frac{1}{x} \int _{0}^{x} \ln f(t) \,dt \biggr\} \,dx \leq e^{\alpha+1} \int_{0}^{\infty}f(x) x^{\alpha}\,dx. $$

The discrete version of inequality (1.3) is known as Carleman’s inequality [5]. Equation (1.3) is a special case of (1.5), and (1.5) can be regarded as a special case of (1.4).

The goal of this note is to complete the following diagram by finding “(2.1)”, which gives (1.2) in the same manner as (1.4) gives (1.5) as a special case.
 This will be done in Sect. 2.1.

## An inequality of Carleson type

There are a lot of different ideas in the literature for the proof of Hardy’s inequality. With the special property of the mean $\frac{1}{x} \int_{0}^{x} f(t) \,dt $, the proof of (1.1) or (1.2) should depend on the convexity of the function $x^{p}, p>1$. See [2] and the references therein.

### $L^{p}$ version inequality

The following theorem is a straightforward consequence of Minkowski’s inequality which comes from the convexity. It verifies Hardy’s inequality (1.2) as we shall see in Sect. 3.1.

#### Theorem 2.1

*Let* $\max\{1, \alpha+1\}< p<\infty$. *Let* *F* *be a nonnegative increasing concave function defined on* $[0, \infty)$ *with* $F(0)=0$. *If* $x^{\alpha-p} F(x)^{p} \in L^{1}(0, \infty)$, *then*

2.1 $$ \int_{0}^{\infty}\biggl(\frac{F(x)}{x} \biggr)^{p} x^{\alpha}\,dx\le \biggl( \frac{p}{p-1-\alpha} \biggr)^{p} \int_{0}^{\infty}\bigl[F'(x) \bigr]^{p} x^{\alpha}\,dx. $$

#### Proof

Fix $k>1$ for a moment. A simple change of variable shows for $A>0$ that

2.2 $$ \begin{aligned}[b] \frac{1}{k^{p}} \int_{0}^{A} x^{\alpha-p} F(kx)^{p} \,dx & = \frac{1}{k^{\alpha +1}} \int_{0}^{kA} x^{\alpha-p} F(x)^{p} \,dx \\ &\ge \frac{1}{k^{\alpha+1}} \int_{0}^{A} x^{\alpha-p} F(x)^{p} \,dx. \end{aligned} $$

By concavity,

2.3 $$ F(kx) \le F(x) + (k-1)x F'(x) $$

and

2.4 $$ 0 \le F'(x) \le\frac{F(x)}{x} $$

for almost every $x \in(0, \infty)$. Inequalities (2.2), (2.3), and Minkowski’s inequality give

2.5 $$ \begin{aligned}[b] & \biggl\{ \frac{1}{k^{\alpha+1}} \int_{0}^{A} x^{\alpha-p} F(x)^{p} \,dx \biggr\} ^{\frac{1}{p}} \le \biggl\{ \frac{1}{k^{p}} \int_{0}^{A} x^{\alpha-p} F(kx)^{p} \,dx \biggr\} ^{\frac{1}{p}} \\ &\quad \le \frac{1}{k} \biggl\{ \int_{0}^{A} x^{\alpha-p} \bigl[F(x) + (k-1)xF'(x) \bigr]^{p} \,dx \biggr\} ^{\frac{1}{p}} \\ &\quad \le \frac{1}{k} \biggl[ \biggl\{ \int_{0}^{A} x^{\alpha-p} F(x)^{p} \,dx \biggr\} ^{\frac{1}{p}} + \biggl\{ \int_{0}^{A} x^{\alpha-p}(k-1)^{p}x^{p} \bigl[F'(x) \bigr]^{p} \,dx \biggr\} ^{\frac{1}{p}} \biggr] \\ &\quad = \frac{1}{k} \biggl\{ \int_{0}^{A} x^{\alpha-p} F(x)^{p} \,dx \biggr\} ^{\frac{1}{p}} + \frac{k-1}{k} \biggl\{ \int_{0}^{A} x^{\alpha} \bigl[F'(x) \bigr]^{p} \,dx \biggr\} ^{\frac{1}{p}} . \end{aligned} $$

By the integrability hypothesis $x^{\alpha-p} F(x)^{p} \in L^{1}(0, \infty )$ and (2.4), the last two integrals are bounded. Thus, it follows from (2.5) that

2.6 $$ \biggl\{ \int_{0}^{A} x^{\alpha-p} F(x)^{p} \,dx \biggr\} ^{\frac{1}{p}} \le C(k) \biggl\{ \int_{0}^{A} x^{\alpha} \bigl[F'(x) \bigr]^{p} \,dx \biggr\} ^{\frac{1}{p}} , $$

where

$$C(k) =\frac{k-1}{k} \frac{1}{k^{-{\frac{\alpha+1}{p}}} -k^{-1} } . $$

Note that $C(k) \to\frac{p}{p-\alpha-1}$ as $k\to1$. Now, letting $k\to1$ and $A\to\infty$ we have (2.1). □

### Remarks

1. The point of Theorem 2.1 is
  $$\int_{0}^{A} \biggl(\frac{F(x)}{x} \biggr)^{p} x^{\alpha}\,dx \sim \int_{0}^{A} \bigl[F'(x) \bigr]^{p} x^{\alpha}\,dx. $$
  In fact, we have
  2.7 $$ \int_{0}^{A} \bigl[F'(x) \bigr]^{p} x^{\alpha}\,dx \le \int_{0}^{A} \biggl(\frac {F(x)}{x} \biggr)^{p} x^{\alpha}\,dx\le \biggl( \frac{p}{p-1-\alpha} \biggr)^{p} \int_{0}^{A} \bigl[F'(x) \bigr]^{p} x^{\alpha}\,dx $$
  by (2.4) and (2.6).
2. The conditions of Theorem 2.1 seem to be rather complicated so that one may suspect the existence of such a *F*. We give a simple example: Let $\alpha, \beta$ be chosen to be $\alpha-p+1<0$, $0<\beta\le p$, and $\alpha-p+\beta+1 >0$. Take $F(x) = x^{\beta/p}$ for $0\le x \le1$ and $F(x)=1$ for $1< x<\infty$. Then *F* is nonnegative increasing concave and $F(0)=0$. A simple calculation shows
  $$\begin{aligned} &\int_{0}^{\infty}\biggl(\frac{F(x)}{x} \biggr)^{p} x^{\alpha}\,dx = \int_{0}^{1} x^{\alpha-p+\beta} \,dx + \int_{1}^{\infty}x^{\alpha-p} \,dx =\frac{1}{\alpha -p+\beta+1} - \frac{1}{\alpha-p+1}, \\ &\int_{0}^{\infty}\bigl[F'(x) \bigr]^{p} x^{\alpha}\,dx = \int_{0}^{1} \biggl( \frac {\beta}{p} x^{\frac{\beta}{p} -1} \biggr)^{p} x^{\alpha}\,dx = \biggl( \frac {\beta}{p} \biggr)^{p}\frac{1}{\alpha-p + \beta+1}, \end{aligned}$$
  whence we have (2.1) (and (2.7) with $A=\infty$).
  On the other hand, the example $F(x) = 0$ for $0\le x \le1$ and $F(x)=1$ for $1< x<\infty$ shows that (2.1) fails if the concavity hypothesis is omitted.
3. Note that a concave function on an interval is absolutely continuous on each closed subinterval. While absolute continuity (on a closed interval) is equivalent to be an indefinite integral. So, the concavity of *F* with $F(0)=0$ in Theorem 2.1 implies
  $$F(x) = \int_{0}^{x} f(y) \,dy \quad\text{for some } f , $$
  which implies the existence of $F'(x)$ almost everywhere and $f=F'$ decreasing.

## Relations between inequalities

In this section, we explain the relationship between inequalities (1.1)–(1.5) and (2.1).

### Hardy’s inequality (1.2) follows from (2.1)

It is simple to see that Hardy’s inequality (1.2) follows immediately from our Theorem 2.1.

First assume $\alpha=0$ and show (1.1). We may assume $f\in L^{p}(0, \infty)$. Let *f̃* be the decreasing rearrangement of *f*:

$$\widetilde{f}(t) =\inf \bigl\{ \lambda\ge0 : \bigl\vert \bigl\{ x : f(x)> \lambda\bigr\} \bigr\vert < t \bigr\} , \quad0\le t < \infty. $$

Then *f̃* is decreasing and

$$\int_{0}^{\infty}f(y) \,dy = \int_{0}^{\infty}\widetilde{f}(y) \,dy, \qquad \int_{0}^{x} f(y) \,dy \le \int_{0}^{x} \widetilde{f}(y) \,dy. $$

Hence it is sufficient to show (1.1) under the hypothesis that *f* is decreasing. Let *f* be decreasing and $F(x) = \int_{0}^{x} f(y) \,dy$. Then $F(0)=0$, $F(x) \ge0$ and $F'(x) = f(x)$ almost everywhere. Since $F'=f$ is nonnegative decreasing, *F* is increasing and concave. Thus, applying Theorem 2.1 with $\alpha=0$, we obtain (1.1).

Next, for general *f* and *α*, (1.2) follows from (1.1) by a simple change of variable: setting

$$g (y) = f \bigl(y^{\frac{p-1}{p-1-\alpha}} \bigr) y^{\frac{\alpha }{p-1-\alpha}}, \quad0< y< \infty, $$

it is straightforward to see

$$\int_{0}^{\infty}\biggl( \frac{1}{x} \int_{0}^{x} f(y) \,dy \biggr)^{p} x^{\alpha}{\,dx} = \biggl( \frac{p-1}{p-1-\alpha} \biggr)^{p+1} \int_{0}^{\infty}\biggl( \frac{1}{x} \int_{0}^{x} g(y) \,dy \biggr)^{p} {\,dx} $$

and

$$\int_{0}^{\infty}f(x)^{p} x^{\alpha}\,dx = \frac{p-1}{p-1-\alpha} \int_{0}^{\infty}g(x)^{p} \,dx , $$

whence

$$\begin{aligned} \int_{0}^{\infty}\biggl( \frac{1}{x} \int_{0}^{x} f(y) \,dy \biggr)^{p} x^{\alpha}{\,dx} &= \biggl( \frac{p-1}{p-1-\alpha} \biggr)^{p+1} \int_{0}^{\infty}\biggl( \frac{1}{x} \int_{0}^{x} g(y) \,dy \biggr)^{p} {\,dx} \\ &\le \biggl( \frac{p-1}{p-1-\alpha} \biggr)^{p+1} \biggl( \frac {p}{p-1} \biggr)^{p} \int_{0}^{\infty}g(x)^{p} \,dx \\ & = \biggl( \frac{p-1}{p-1-\alpha} \biggr)^{p+1} \biggl( \frac {p}{p-1} \biggr)^{p} \frac{p-1-\alpha}{p-1} \int_{0}^{\infty}f(x)^{p} x^{\alpha}\,dx \\ & = \biggl( \frac{p}{p-1-\alpha} \biggr)^{p} \int_{0}^{\infty}f(x)^{p} x^{\alpha}\,dx. \end{aligned}$$

### Inequality (1.5) follows from Carleson’s inequality (1.4)

It is also simple to see that inequality (1.5) follows from Carleson’s inequality (1.4).

Let *f̃* be the decreasing rearrangement of *f*, and let $F(x) = -\int_{0}^{x} \ln\widetilde{f}(y) \,dy$. Then $F(0)=0$, and $F'(x) = -\ln\widetilde{f}(x)$ almost everywhere. Since *f̃* is decreasing, $F'$ is increasing, whence *F* is convex by an elementary calculation. Thus, applying Theorem 1.2 with $\alpha=0$, we obtain

3.1 $$ \begin{aligned}[b] \int_{0}^{\infty}\exp \biggl\{ \frac{1}{x} \int_{0}^{x} \ln f(t) \,dt \biggr\} \,dx & \leq \int_{0}^{\infty}\exp \biggl\{ \frac{1}{x} \int_{0}^{x} \ln\widetilde {f}(t) \,dt \biggr\} \,dx \\ & \leq e \int_{0}^{\infty}\widetilde{f}(x) \,dx = e \int_{0}^{\infty}f(x) \,dx. \end{aligned} $$

Next, let $g(x) = x^{\alpha}f(x)$ and *g̃* be the decreasing rearrangement of *g*. Then, by (3.1) with *g* in place of *f*,

$$\begin{aligned} \int_{0}^{\infty}x^{\alpha}\exp \biggl\{ \frac{1}{x} \int_{0}^{x} \ln f(t) \,dt \biggr\} \,dx &= e^{\alpha}\int_{0}^{\infty}\exp \biggl\{ \frac{1}{x} \int_{0}^{x} \ln \bigl( {t^{\alpha}} {f(t)} \bigr) \,dt \biggr\} \,dx \\ &=e^{\alpha}\int_{0}^{\infty}\exp \biggl\{ \frac{1}{x} \int_{0}^{x} \ln g(t) \,dt \biggr\} \,dx \\ &\leq e^{\alpha}\int_{0}^{\infty}\exp \biggl\{ \frac{1}{x} \int_{0}^{x} \ln \widetilde{g}(t) \,dt \biggr\} \,dx \\ &\leq e^{\alpha+1} \int_{0}^{\infty}\widetilde{g}(x) \,dx = e^{\alpha+1} \int_{0}^{\infty}f(x) x^{\alpha}\,dx. \end{aligned}$$

### Remarks

1. The limit case $p\to\infty$ of (1.1) (replacing *f* by $f^{\frac{1}{p}}$ first) represents (1.3), and the same limit case of (1.2) represents (1.5). We can summarize the relations between (1.1)∼(1.5) and (2.1) as the diagram at the end of Sect. 1.
2. Minkowski’s inequality appeared in 1896 [6] (see Minkowski’s posthumous [7]) and was expanded to the integral form in the context of $L^{p}$ space in 1910 [8], while Hardy’s inequality appeared, and was verified and extended in 1920 [9], 2025 [10], and 1928 [1], respectively.

## Conclusion

From the observation that the Polya–Knopp inequality is a limit version of Hardy’s inequality and Carleson’s inequality verifies Polya–Knopp inequality as a special case, an inequality verifying Hardy’s inequality by the same pattern was called for. In Theorem 2.1, the main result of this article, we give such an inequality: inequality (2.1).

A simple application of Minkowski’s inequality proved Theorem 2.1. In view of Section 3.3 Remarks (2), it seems that there should have been an easy proof of Hardy’s inequality in terms of Minkowski’s inequality early in the literature.
